# PDTC Alleviates Depressive Symptoms and Colon Tissue Injury via Inhibiting NO Overproduction in CUMS Rats

**DOI:** 10.3389/fnins.2019.01327

**Published:** 2019-12-17

**Authors:** Lejin Yang, Hui Chen, Dongdong Wang, Shuping Nie, Jinge Du, Ming Lu

**Affiliations:** ^1^Department of Psychology, Qilu Hospital of Shandong University, Jinan, China; ^2^Clinical Epidemiology Unit, Qilu Hospital of Shandong University, Jinan, China; ^3^Brain Laboratory, Qilu Hospital of Shandong University, Jinan, China

**Keywords:** chronic and unpredictable mild stress, spinal cord, colon, depression, NF-κB, nitric oxide

## Abstract

**Background:**

The accumulated evidence demonstrates that stress plays an important role in the pathogenesis of depression that is associated with intestinal dysfunctions. However, the mechanisms remain unresolved.

**Methods:**

A total of 40 male Wistar rats were obtained and randomly divided into four equal-sized group: control, PDTC + chronic and unpredictable mild stress (CUMS), FLX + CUMS, and CUMS. Western blotting and qRT-PCR were used to examine the levels of nitric oxide (NO), nuclear factor kappa beta (NF-κB), inducible nitric oxide synthase (iNOS), and iNOS mRNA in spinal cord L1-2 and colon.

**Key Results:**

Chronic and unpredictable mild stress increased the serum CORT level, decreased body weight and sucrose preference, and altered OFT performance, while increased levels of NO, iNOS mRNA, iNOS and NF-κB protein in colon and spinal cord were accompanied by histopathological changes in colon. Pretreatment with an NF-κB inhibitor, pyrrolidine dithiocarbamate (PDTC), reversed these effects. Fluoxetine failed to prevent NO increase in both spinal cord and colon, while the iNOS protein level, although not statistically significantly increased compared to control, was not decreased compared to CUMS. Also, fluoxetine failed to prevent histological changes.

**Conclusion:**

In conclusion, the NF-κB/iNOS pathway may be involved in the mechanism of CUMS-induced depressive-like behavior and colon tissue injury.

## KEY POINTS

- We investigated the role of NF-κB in the development of stress in rat colon and spinal cord following chronic and unpredictable mild stress (CUMS).

- Chronic and unpredictable mild stress increased the serum CORT level, decreased body weight and sucrose preference, and altered OFT performance, while increased levels of NO, iNOS mRNA, iNOS and NF-κB protein in colon and spinal cord were accompanied by histopathological changes in colon. Pretreatment with an NF-κB inhibitor, PDTC, and reversed these effects. Fluoxetine failed to prevent NO increase in both spinal cord and colon, while the iNOS protein level, although not statistically significantly increased compared to control, was not decreased compared to CUMS. Also, fluoxetine failed to prevent histological changes.

- The NF-κB/iNOS pathway might be involved in the mechanism of CUMS-induced colon tissue injury. Four weeks of pretreatment with fluoxetine could not inhibit the weight loss in depressive rats and colon tissue damage that are induced by excessive production of iNOS-derived NO.

## Introduction

It has been documented that neuropsychiatric disorders are closely associated with dysfunctions of the gut (Zhao et al., 2018). Ample clinical studies also show that the prevalence of mental disorders, especially depression and anxiety, in patients with gastrointestinal (GI) symptoms is approximately 80% (Kurina et al., 2001). Meanwhile, cognitive-behavioral therapy and antidepressants have been confirmed to alleviate the symptoms of patients with GI disorders (Wiley et al., 2016). It is well known that stress is an important factor in the genesis of neuropsychiatric disorders (Gulati et al., 2015). Furthermore, stress can regulate gut permeability, which in turn increases gram-negative bacteria translocation into the mesenteric lymph nodes and blood and causes immune-inflammatory activity (Anderson, 2018). However, the mechanisms of dysfunctions in the gut that are induced by stress-associated conditions are poorly understood.

Stress exposure increases the production of NO. It is well established that NO plays a crucial role in the regulation of stress-induced neurobehavioral, immunological, GI, endocrinal, and biochemical responses to stress (Pal et al., 2011; Gulati et al., 2015). Studies have demonstrated that overproduction of NO induced by iNOS is involved in tissue injury in many GI diseases (Kimura et al., 1997) and that the inhibition of iNOS can ameliorate the gut tissue damage (Menchen et al., 2001). Furthermore, chronic stress can increase the likelihood of relapse in patients with quiescent inflammatory bowel disease (Graff et al., 2009). On the other hand, NO is a kind of gas molecule that has been reported to play a major role in the pathogenesis of stress-related disorders (Gulati et al., 2015). Evidence is accumulating that chronic stress exposure can cause overproduction of NO, resulting in disruption of hypothalamic-pituitary-adrenal (HPA) axis activity (Gadek-Michalska et al., 2019). The activation of the HPA axis is one of the central physiological mechanisms involved in the stress response (McEwen et al., 2015). The dysfunction of the HPA axis plays an important etiological role in the development of depression (Dantzer, 2006; Zhou et al., 2011). Furthermore, a recent study also found chronic or acute stress-induced iNOS-derived NO overproduction in both the hippocampus and prefrontal cortex of rats (Gadek-Michalska et al., 2019). Accordingly, it has been suggested that NOS inhibition be developed as a novel antidepressant strategy (Montezuma et al., 2012). Taken together, these observations have prompted the hypothesis that an impaired NO pathway may play an important role in the progress of depression that is associated with gut dysfunction.

Previous studies found that stress could increase iNOS expression in the cerebral cortex via the NF-κB pathway (Zlatkovic and Filipovic, 2013). It is well known that NF-κB is sequestered in the cytoplasm complexed to its inhibitor IκB under basal conditions. In response to stress, phosphorylated IκB molecules undergo polyubiquitination and subsequent degradation, leading to the translocation of NF-κB to the nucleus, and activation of the expression of target genes, such as *iNOS* (Baud and Karin, 2009).

It is worth mentioning that the GI tract maintains an extensive intrinsic nervous system. The intrinsic enteric nervous system (ENS) can exert influences on the intestinal tract even when it is disconnected from the CNS (Bajic et al., 2018). The ENS is controlled by extrinsic innervation from the lower spinal cord, where sympathetic fibers suppress contraction of the colonic musculature, and parasympathetic fibers conversely facilitate colon motility, consequently affecting immune-, mucosa-, and microflora-related alterations (Camilleri and Ford, 1998; Mayer, 2011). Ample clinical studies demonstrate that spinal cord injury can increase intestinal permeability and cause intestinal dysfunction (Kigerl et al., 2016). In particular, iNOS is considered to be a marker of M1 macrophages, and the infiltration of M1 macrophages is considered to be the main cause of secondary injury cascade (Klingener et al., 2014). In the GI tract, NO is widely regarded to regulate several functions in both physiological and pathological states, including maintaining the integrity of GI mucosa, smooth muscle function, or mucosal inflammation (Wallace, 2019). NO is synthesized by neuronal NO synthase (nNOS), endothelial NOS (eNOS), and iNOS in the different cell types, and all of the NOS isoforms are present in mRNA and protein in the enteric neurons (Bagyanszki et al., 2011). iNOS-derived NO is released in large quantities during inflammation, which may be cytotoxic to enteric neurons (Bodi et al., 2019). However, few studies have addressed the effects of the iNOS/NO pathway in the spinal cord on GI function under stress conditions.

In the present study, we aimed to investigate the effects of PDTC, an inhibitor of NF-κB, and fluoxetine, as a positive control, on behavioral changes, body weight, and colon tissue as well as to explore the mechanism of chronic and unpredictable mild stress (CUMS)-induced colon tissue injury in depressive rats.

## Materials and Methods

### Animal Preparation

A total of 40 male Wistar rats (2 months, 180–220 g) were obtained from the Animal Experiment Centre of Shandong University. All animals were maintained at an ambient temperature of 20 ± 4°C and 36–60% relative humidity in a light-cycled room (12:12 h). Food and water were given *ad libitum* unless otherwise noted. They were allowed to habituate to the controlled environment for 1 week before experimentation. This study was approved by Shandong University Animal Care and Use Committee. All of the animal experiments were performed according to the institutional guidelines for animal care and use.

A total of 40 animals were randomly divided into four equally sized groups. The control rats received a daily intraperitoneal injection of sterile saline. All other rats received a daily intraperitoneal injection of pyrrolidine dithiocarbamate (PDTC, Sigma Company, 100 mg/kg) (Xia et al., 2016) or fluoxetine (Eli Lilly and Co., 10 mg/kg) (Lee et al., 2012), or sterile saline 30 min prior to CUMS exposure. The total treatment period was 28 days. The animals in the control group were left undisturbed in the home cages, while others were subjected to 28 days of CUMS according to Katz’s model with a minor modification (Katz et al., 1981). Rats were exposed to one of the following stressors randomly every day: fasting (24 h), water deprivation (24 h), tail clamping (1 min), day-night reversal (12 h/12 h), noise exposure (1 h, 1500 Hz, 92 dB), restraint stress (1 h), or shaking (15 min). No same stressor was applied continuously for 2 days.

### Body Weight

Body weights were measured every day throughout the CUMS protocol.

### Open Field Test (OFT)

After CUMS exposure, OFT was conducted between 7:30 am and 11:30 am in a quiet room. The floor of the apparatus was divided into 5 × 5 equal areas. Each animal was placed in the central square and then tested for 5 min. The following indices were recorded: time in central area, rearing number (standing on two hind limbs), square-crossing number (with three paws in one square), and grooming number. The apparatus was cleaned with 5% alcohol after the test.

### Sucrose Preference Test (SPT)

Rats were trained for adaptation to both tap water and a 1% sucrose solution before the test. In order to eliminate the effect of position, we counterbalanced bottles across the right and left sides of the cages throughout the experiment. All rats were allowed to consume the sucrose solution for 1 h after the food and water had been removed for a period of 23 h. Results were recorded by reweighing pre-weighed bottles of test solution. The percentage preference (PP) for sucrose was calculated by the following formula: PP = (sucrose intake/total fluid intake) × 100%.

### Measurement of NO Content

The proximal colon and spinal cord at L1-2 tissues were weighed and homogenized in 1:9 w:v in 0.9% saline. The homogenates were centrifuged at 1000 r/min for 5 min at 4°C, and the supernatant was taken for NO assay. The level of NO was determined spectrophotometrically by measuring total nitrate plus nitrite (NO_3_^–^ plus NO_2_^–^) and the stable end products of NO metabolism. The procedure in which nitrate was enzymatically converted into nitrite by the enzyme nitrate reductase was followed by quantization of nitrite using Griess reagent at an absorbance of 550 nm. NO level was expressed as μmol/g protein.

### Serum Corticosterone Assay

Serum corticosterone (CORT) levels were measured using the corticosterone ELISA kit following the manufacturer’s protocol. Serum was obtained after the blood was held for 1 h at 20°C and stored at −80°C until assayed. The plates were read at 405 nm using a BioRad 3560 ELISA plate reader. The results were expressed as ng/ml compared with standards consisting of either OVA-specific serum from hyperimmunized mouse serum.

### Western Blotting

Tissue preparation of colon and spinal cord: A segment of proximal colon (1 cm from caecum) and spinal cord at L1-2 tissues were collected (Zhao et al., 2017) and then stored at −80 °C until assayed. First, the frozen tissues were homogenized in cytoplasmic extraction reagent A CERA (0.2 ml) (BioTeke Corporation, Beijing, China). The homogenates were centrifuged at 15,000 rpm for 10 min at 4°C. The supernatant containing cytoplasmic proteins was collected and stored at −80°C to measure the level of cytosolic IκB. Second, the sediment was dissolved in phenylmethanesulfonylfluoride buffer (0.05 ml) and diluted by the same volume of nuclear extraction reagent B (NER B), then rotated for 30 min at 4°C. The supernatant was collected as nuclear extracts and stored at −80°C after centrifugation at 15,000 rpm for 10 min at 4°C to measure the level of nuclear NF-κB. Third, a segment of proximal colon or spinal cord L1-2 tissue was weighed and homogenized (1:8 w/v) in RIPA solution (Beyotime Institute of Biotechnology, Shanghai, China) for measurement of iNOS levels.

Experimental procedure: Briefly, the extracted homogenates were collected and measured. After separation by electrophoresis, the proteins were transferred onto polyvinylidene difluoride (PVDF membrane, Millipore) in transfer buffer. The membranes were then blocked for 1 h in TTBS containing 5% dried skimmed milk powder and incubated overnight with the primary antibody (iNOS, 1:1000; β-actin, 1:5000; NF-κB,1:800; C-Jun, 1:1000, Santa Cruz, CA, United States). After that, membranes were incubated for 1 h at room temperature with horseradish peroxidase (HRP)-conjugated secondary antibodies (iNOS, 1:5000; β-actin, 1:10000, Peroxidase-Conjugated AffiniPure IgG; NF-κB,1:10000; C-Jun, 1:15000, Zhongshan Jinqiao Bio., China). The images of specific antigen bands were quantified by using Scion Image software (Version Alpha 4.0.3.2, Scion Corporation, Frederick, MD, United States). The western blot results were quantified using Scion Image software; the background band was subtracted, and the band was expressed as relative protein amounts compared to β-actin (cytoplasmic protein) or c-jun (nuclear protein).

### qRT-PCR

Briefly, total RNA was extracted using Trizol reagent according to the manufacturer’s instructions. Quantitative RT-PCR was performed with an ABI Prism 7500 sequence detection system (Applied Biosystems) using the Quantitative SYBR Green PCR Kit (TIANGEN, China) according to the manufacturer’s instructions. qRT–PCR was performed in triplicate using region-specific primers for iNOS Forward:5′-CCT CCT CCA CCC TAC CAA GT-3′ and Reverse: 5′-CAC CCA AAG TGC TTC AGT CA-3′; NF-κB Forward:5′-TTG GAG CGA GTT GTG GAT TG -3′; and Reverse:5′-GAA GCC TCT TGT CTT TGA CCC-3′; β-actin Forward:5′-GAC AGG ATG CAG AAG GAG ATT ACT-3′; and Reverse:5′-TGA TCC ACA TCT GCT GGA AGG T-3′.

### Histological Analysis

Colonic tissues were fixed with 4% paraformaldehyde and embedded in paraffin. Sections (5 μm) were stained using hematoxylin and eosin (H&E) staining.

### Statistical Analysis

The data were analyzed by one-way ANOVA, followed by the Student-Newman-Keuls *post hoc* test. All values were represented as mean ± SE. Statistical significance was accepted at *p* < 0.05.

## Results

### Effects of PDTC on Body Weight

**FIGURE 1:**
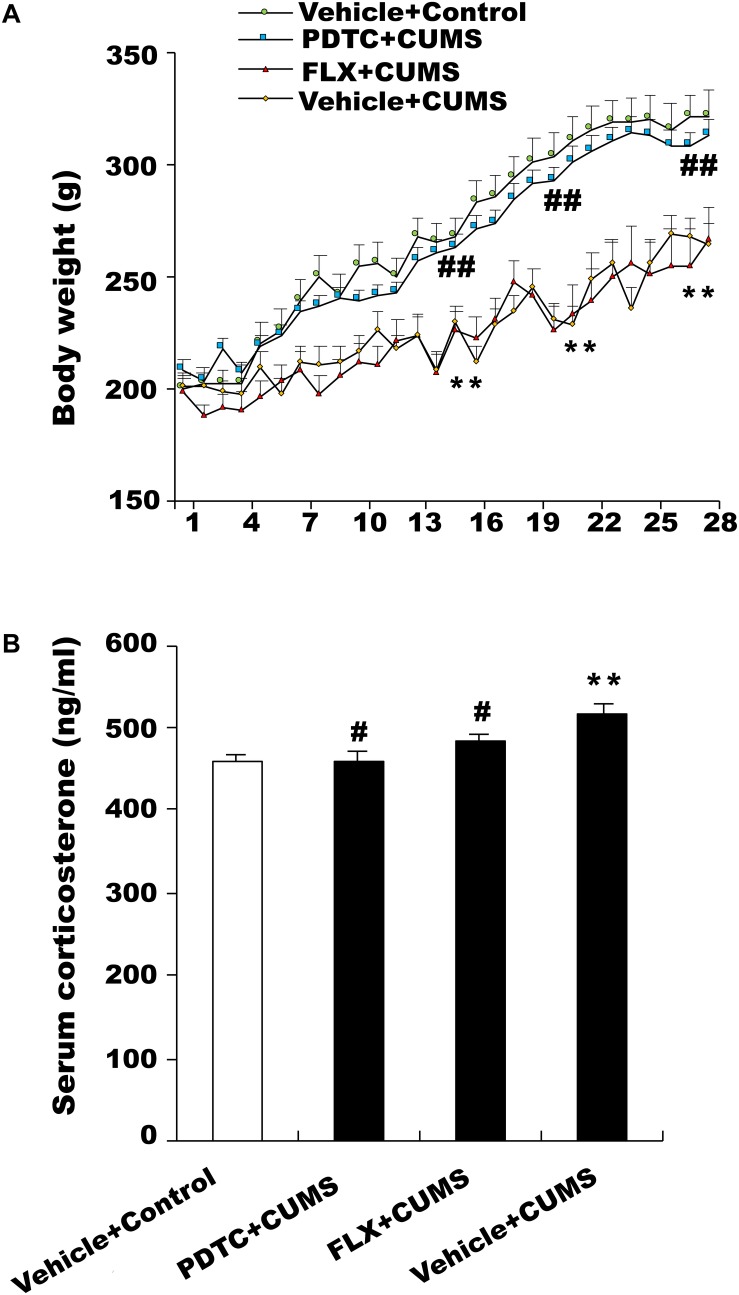
Changes in body weight and serum corticosterone levels in response to chronic and unpredictable mild stress (CUMS). **(A)** Body weight in the CUMS group was significantly lower than in the control group. Pretreatment with pyrrolidine dithiocarbamate (PDTC), but not fluoxetine, reversed CUMS-induced weight loss. **(B)** CUMS rats showed an obviously higher level of serum CORT compared with the control group. PDTC and fluoxetine inhibited CUMS-induced increase in serum CORT. All values are presented as mean ± SEM. *n* = 10 and ^∗∗^*p* < 0.01 versus the control group. ^#^*p* < 0.05 and ^##^*p* < 0.01 versus the CUMS group. FLX, fluoxetine.

During the 28 days of CUMS treatment, the body weights of the rats were examined daily. The results showed that CUMS group rats displayed significantly lower body weight compared to the control group. Pretreatment with PDTC, an NF-κB inhibitor, was able to reverse CUMS-induced decrease in body weight. However, pretreatment with fluoxetine had no effect on the body weight of rats suffering from CUMS (Figure 1A).

### Effects of PDTC on Serum Corticosterone (CORT) Levels

The serum CORT level in CUMS group rats was higher than that of the control group. Both fluoxetine and PDTC pretreatment could inhibit CUMS-induced increase of the serum CORT level in rats (Figure 1B).

### Effects of PDTC on Behavioral Changes in OFT and SPT

**FIGURE 2:**
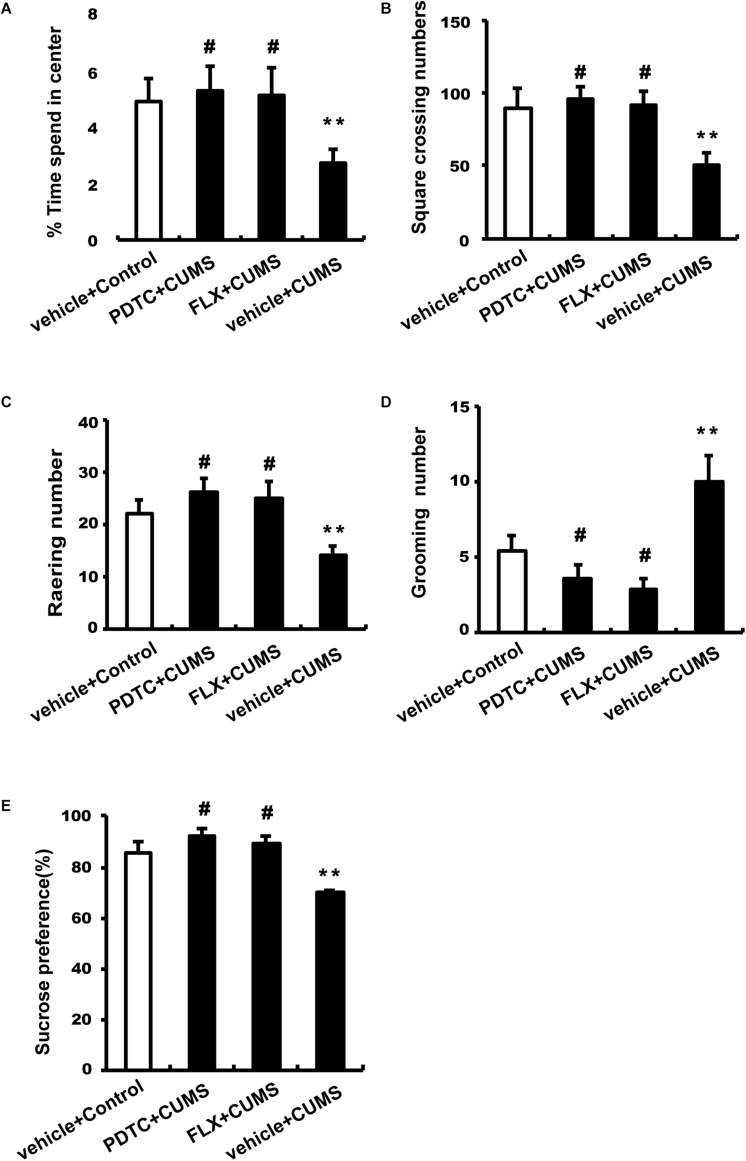
Administration of PDTC and fluoxetine reversed CUMS-induced depression-and anxiety-like behaviors in rats. After CUMS treatment, all rats were subjected to OFT and SPT. Compared to the CUMS group, both PDTC and fluoxetine pretreatment reversed CUMS-induced decrease in the time spent in the center **(A)**, square-crossing number **(B)**, and rearing number **(C)** and increase in grooming number **(D)**. In SPT, the sucrose consumption of CUMS rats decreased significantly, which could be rescued by PDTC or fluoxetine pretreatment **(E)**. All values are presented as mean ± SEM. *n* = 10 and ^∗∗^*p* < 0.01 versus the control group. ^#^*p* < 0.05 versus the CUMS group. FLX, fluoxetine.

After CUMS treatment, rats were subjected to OFT and SPT to evaluate depressive and anxiety-like behaviors. CUMS group rats spent less time in the center and had an increase in square-crossing number, rearing number, and grooming number compared to control group rats, which could be reversed by both PDTC and fluoxetine pretreatment before CUMS (Figures 2A–D). CUMS group rats showed significantly decreased sucrose consumption compared to the control group, while PDTC or fluoxetine pretreatment could significantly rescue the deficit of sucrose consumption induced by CUMS in rats (Figure 2E).

### Effects of PDTC on Colon Tissue Damage

**FIGURE 3:**
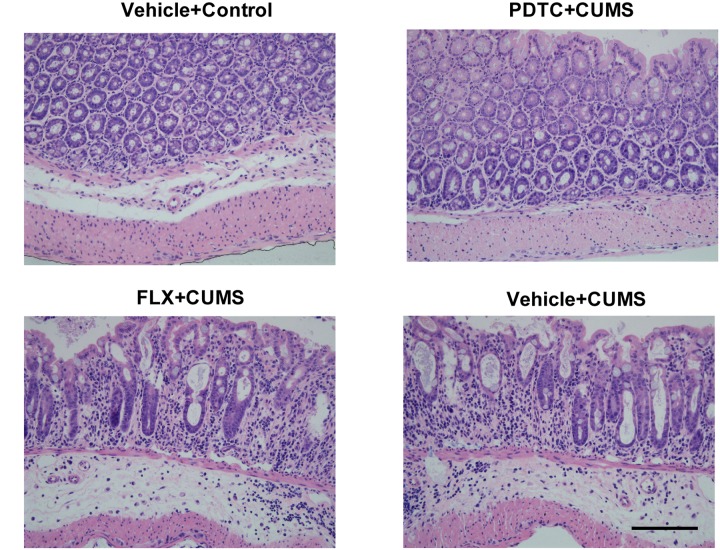
Pyrrolidine dithiocarbamate pretreatment attenuated CUMS-induced colonic tissue damage in rats. HE staining showed that CUMS caused severe cellular damage and crypt necrosis in colon tissues, which could not be rescued by fluoxetine pretreatment. However, PDTC pretreatment could significantly attenuate CUMS-induced colon tissue damage.

HE staining showed that CUMS induced severe cellular damage and crypt necrosis in the colonic tissues in rats, which could not be rescued by fluoxetine pretreatment. However, PDTC pretreatment before CUMS could significantly attenuate CUMS-induced colon tissue damage (Figure 3).

### Effects of PDTC on the Production of NO in Colon and Spinal Cord

**FIGURE 4:**
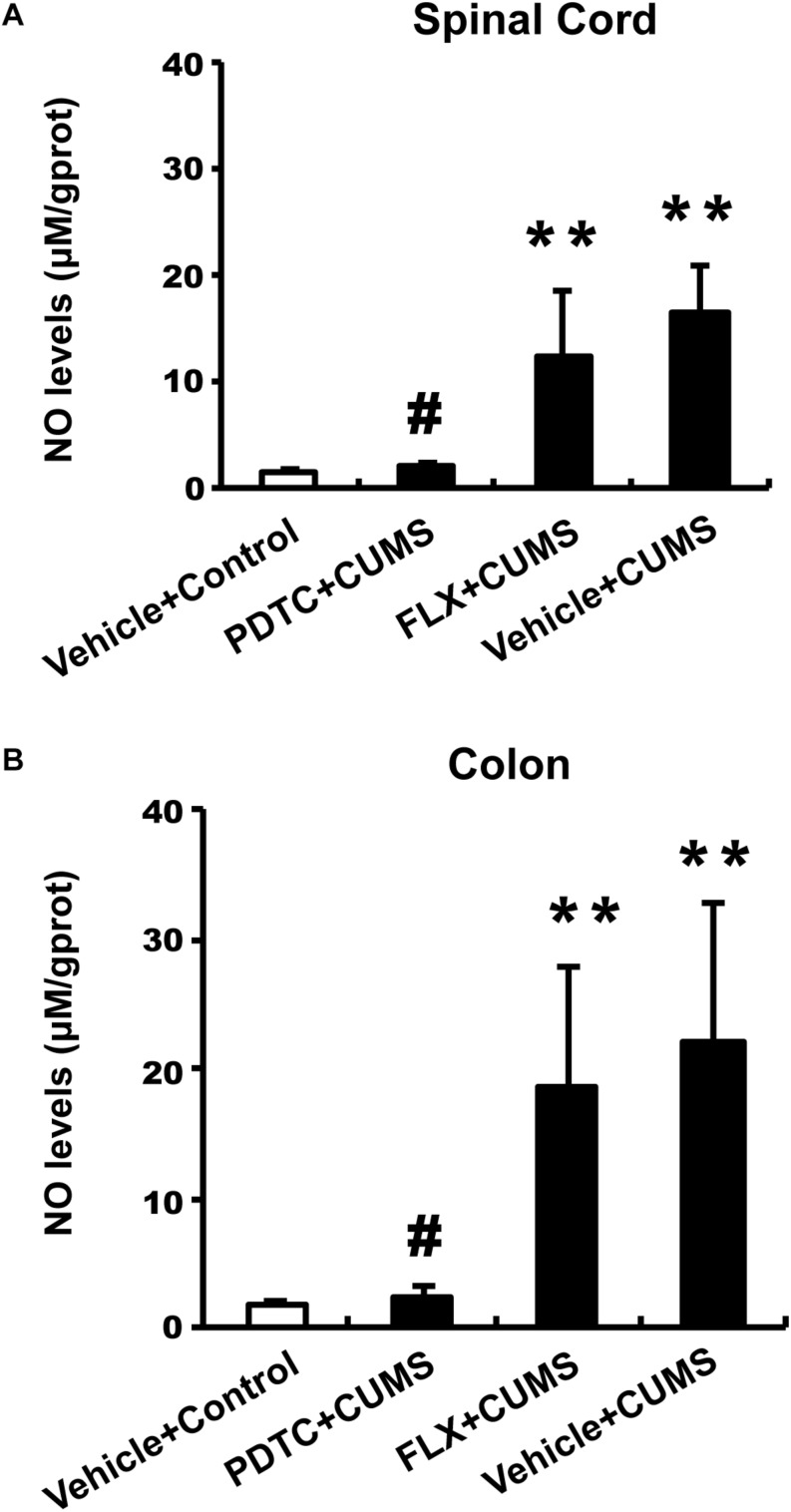
Pyrrolidine dithiocarbamate but not fluoxetine inhibited CUMS-induced production of NO in both spinal cord and colon. The CUMS group and fluoxetine-pretreatment group showed significantly higher levels of NO content in both the spinal cord **(A)** and colon **(B)** compared to the control group. PDTC pretreatment significantly decreased the level of NO content compared to the CUMS group in both spinal cord **(A)** and colon **(B)**. All values are presented as mean ± SEM. *n* = 10 and ^∗∗^*p* < 0.01, versus the control group. ^#^*p* < 0.05 versus the CUMS group. FLX, fluoxetine.

We examined the NO content in both the colon and spinal cord L1-2. The CUMS group and fluoxetine-pretreatment group rats showed significantly higher levels of NO content in both the spinal cord (Figure 4A) and colon (Figure 4B) compared to the control group. However, intraperitoneal administration of PDTC before CUMS significantly decreased the level of NO content compared to the CUMS group in both the spinal cord (Figure 4A) and colon (Figure 4B). The results indicated that CUMS was able to enhance the production of NO in both spinal cord and colon and that inhibition of NF-κB signaling could prevent CUMS-induced production of NO, suggesting that the excessive production of NO is downstream of NF-κB signaling in response to CUMS.

### Effects of PDTC on iNOS in Colon and Spinal Cord

**FIGURE 5:**
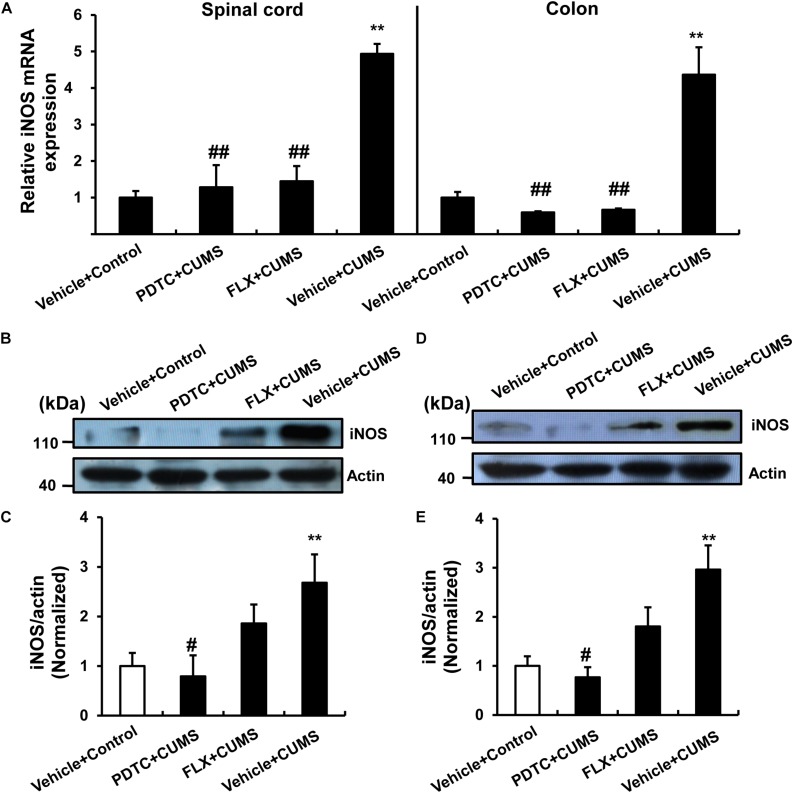
PDTC prevented CUMS-induced expression of iNOS in both spinal cord and colon. **(A)** CUMS exposure resulted in a significant up-regulation of iNOS mRNA in spinal cord and colon. PDTC pretreatment reversed CUMS-induced increase in iNOS mRNA expression. Pretreatment with PDTC, but not fluoxetine, inhibited CUMS-induced increase in iNOS protein levels in the spinal cord **(B,C)** and colon **(D,E)**. All values are presented as mean ± SEM. *n* = 6 and ^∗∗^*p* < 0.01 versus the control group. ^#^*p* < 0.05 and ^##^*p* < 0.01 versus the CUMS group. FLX, fluoxetine.

As the production of NO depends on the function of iNOS, we next examined the expression of iNOS in response to CUMS or drug pretreatment. PDTC was able to reverse CUMS-induced iNOS mRNA expression in both the spinal cord and colon (Figure 5A) compared to the control group. According to the western blot analysis, we found that PDTC inhibited CUMS-induced increase in the iNOS protein levels in both the spinal cord (Figures 5B,C) and colon (Figures 5D,E).

### Effects of PDTC on NF-κB

**FIGURE 6:**
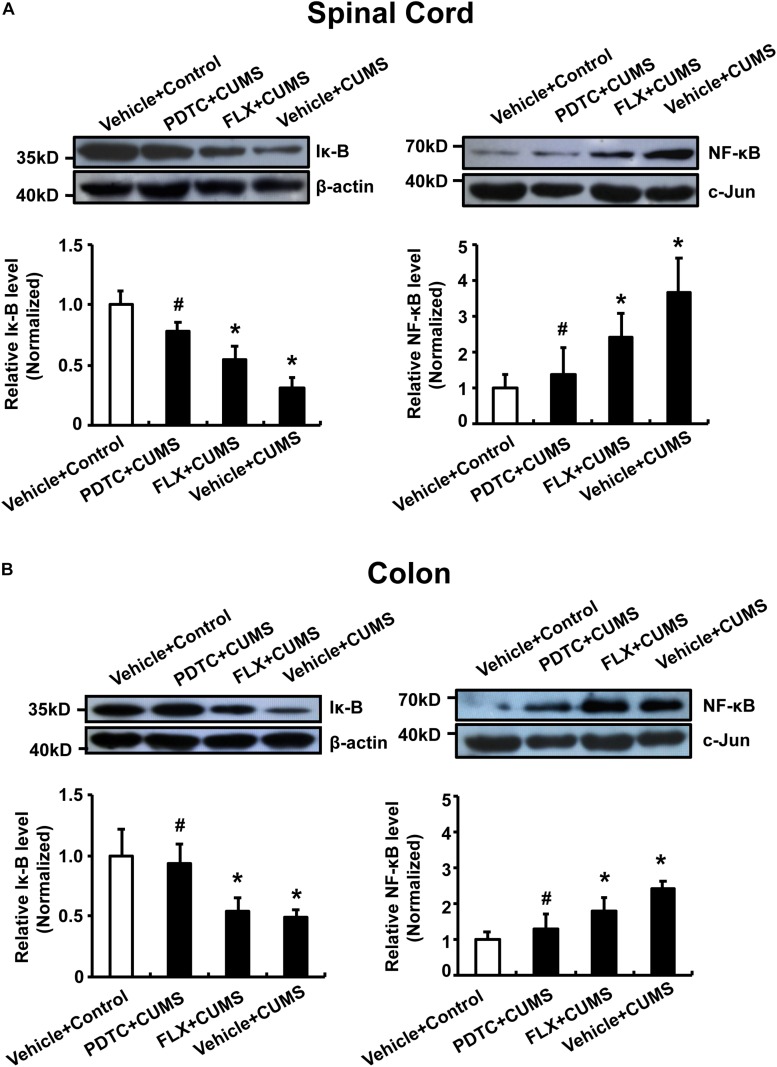
Pyrrolidine dithiocarbamate prevented nuclear translocation of NF-κB in the spinal cord and colon of CUMS rats. CUMS obviously reduced cytosolic IκB levels and increased nuclear NF-κB levels in both spinal cord and colon. Pretreatment with PDTC, but not fluoxetine, prevented CUMS-induced nuclear translocation of NF-κB in spinal cord **(A)** and colon **(B)**. All values are presented as mean ± SEM. *n* = 6 versus the control group. ^#^*p* < 0.05 versus the CUMS group. FLX, fluoxetine.

Further biochemical analysis revealed that CUMS obviously reduced cytosolic IκB levels and increased nuclear NF-κB levels in both spinal cord and colon. CUMS promoted nuclear translocation of NF-κB in both the spinal cord and colon compared to the control group, which could not be reversed by fluoxetine pretreatment. However, intraperitoneal administration of PDTC significantly prevented CUMS-induced nuclear translocation of NF-κB (Figures 6A,B).

## Discussion

Stress has been shown to be associated with both the onset and maintenance of GI symptoms. Furthermore, patients with GI dysfunctions have been found to be more likely to develop psychiatric disorders before the onset of GI symptoms (Mayer et al., 2001; Donald et al., 2017; Aubert et al., 2018). However, the mechanisms underlying stress-gut dysfunctions have not yet been fully elucidated. Our results indicated that 28-day CUMS exposure resulted in depressive and anxiety-like behaviors and HPA-axis dysfunction in rats. Additionally, the CUMS rats also showed significantly lower body weight, severe cellular damage, and crypt necrosis in the colonic tissues. Pretreatment with an NF-κB inhibitor, PDTC, reversed these effects. Furthermore, PDTC also inhibited the overproduction of iNOS-derived NO in both spinal cord L1-2 and colon. This implied that the NF-κB/iNOS pathway might be involved in the pathologic process of CUMS-induced depressive symptoms and colon tissue damage. As expected, fluoxetine pretreatment reversed the behavioral changes and HPA axis dysfunction induced by CUMS. Interestingly, pretreatment with fluoxetine did not inhibit weight loss in depressive rats after 4 weeks of exposure. Moreover, fluoxetine neither alleviated colon damage nor inhibited the higher levels of NO content in either spinal cord or colon. Besides this, fluoxetine also failed to reverse the up-regulation of iNOS and NF-κB. It was speculated that there was insufficient treatment time for it to inhibit the colon tissue injury and weight gain.

In the present study, we employed a CUMS mouse model, as chronic and unpredictable mild stress protocols more accurately reflect the chronic symptoms and mechanisms of mood disorders. This animal model has been accepted to have satisfactory reliability and validity (Forbes et al., 1996; Willner, 2005). After 28-day CUMS exposure, rats showed obviously decreased exploratory activity, increased anxiety level, and less consumption of sweet solution compared with control rats. These results were consistent with previous studies (Jiang and Zhu, 2015). In addition, fluoxetine pretreatment reversed the depressive and anxiety-like behaviors induced by CUMS. However, fluoxetine could not inhibit the trend of CUMS-induced slow weight gain, similar to the previous study. Furthermore, the results of Lijuan Sun et al. also showed that the slow trend of weight gain of fluoxetine-treated rats gradually improved after the fourth week (Sun et al., 2019). A previous study demonstrated that stress could increase iNOS levels in the cerebral cortex through the NF-κB pathway (Zlatkovic and Filipovic, 2013). Furthermore, stress caused the overproduction of iNOS-derived NO, which further led to behavioral changes and HPA axis dysfunction (Gadek-Michalska et al., 2019). Based on these findings, it is suggested that PDTC could influence behavioral changes induced by CUMS.

Neuroimmune-endocrine dysfunction is one of the fundamental mechanisms contributing to the pathophysiology of stress-related disorders (Haj-Mirzaian et al., 2016). The HPA axis, in particular, is crucial for coordinating neuroendocrinal responses to stress that promote survival and homeostasis (Gulati et al., 2015). Furthermore, ample clinical and preclinical studies have confirmed that dysfunction of the HPA axis plays a key etiological role in the pathophysiology of stress-related disorders (Zhou et al., 2011). CUMS exposure activates the HPA axis and is accompanied by continuous serum cortisol (human) or CORT (rodents) secretion. Conversely, a persistent high serum CORT concentration impairs feedback regulation and then induces HPA axis dysfunction (Gulati et al., 2015). On the other hand, the HPA axis mediates stress-induced alterations in GI. For instance, an impaired HPA axis response to environmental stressors is thought to be involved in stress-related adverse effects on the outcome of the gut (Pinto-Sanchez et al., 2015). In addition, stress and altered regulation of CORT have been implicated in intestinal dysfunction and alterations in the gut microbiome (Wiley et al., 2016). Therefore, exploration of the activity of the HPA axis becomes a very important channel for exploring brain-gut interaction. In this study, CORT is considered to be a marker of HPA axis activation (Bachis et al., 2008). CUMS exposure caused the continuous activation of the HPA axis, which in turn led to the release of large quantities of CORT. Compared to the CUMS group, although pretreatment with fluoxetine reduced the serum CORT level and reversed the behavioral change of rats, it failed to improve their colon tissue damage and weight gain. There are two possible mechanisms that could explain these results: first, the continuous activation of the HPA axis only affects the behavioral changes of rats but does not directly affect the changes in the GI tract and body weight induced by CUMS; second, there was not enough time for it to reverse these changes. In contrast, PDTC not only reduced the serum CORT level but also reversed the changes in colon tissues and body weight. This indicates that the NF-κB pathway might be involved in the activation of the HPA axis. Therefore, it is credible that inhibition of NF-κB activation impedes the continuous release of CORT induced by CUMS exposure, which consequently ameliorates the impaired HPA axis response and further affects the colon tissue and body weight.

Previous studies have reported that iNOS-derived NO following chronic stress may induce depressive-like behaviors in mice (Zhou et al., 2007). Our previous study also found that iNOS levels were markedly up-regulated in both the hippocampus and prefrontal cortex of CUMS rats (Yang et al., 2012). In addition, clinical data suggest that an elevated plasma level of NO may be associated with suicide attempts and the severity of depressive symptoms (Talarowska et al., 2012). NO levels are elevated in patients with depression and are regarded to be associated with psychomotor retardation (Akpinar et al., 2013). It is well known that NO plays a significant role in the neurobiology of depression through its modulatory effects on various neurotransmitters (Akyol et al., 2004). Therefore, NO is considered to play a crucial role in the development of depression (Gulati et al., 2015). On the other hand, it has been suggested that excessive iNOS-derived NO levels are associated with many GI diseases (Rivera et al., 2011; Temiz et al., 2016). Interstitial cells of Cajal innervated by nitrergic nerves can increase the production of NO (Lies et al., 2015). The latter is thought to affect colonic permeability in rats (Benabdallah et al., 2008). Moreover, sustained iNOS activation in response to stress exposure results in a surplus of NO, which also undermines colon integrity via the synthesis of peroxynitrite (Gulati et al., 2015). It has been mentioned before that motility and secretion in the colon are controlled by the ENS, which is regulated by extrinsic innervation from the spinal cord (den Braber-Ymker et al., 2017). The spinal cord and intestine interact with each other in a variety of ways (Brierley et al., 2004). Therefore, attempts to study the effects of the spinal cord on the intestine by intrathecal injection of drugs have certain limitations. In the present study, we used intraperitoneal injection of PDTC to study its interaction mechanism (Tegeder et al., 2004). The results indicate that CUMS exposure increased the levels of iNOS-induced NO in both the spinal cord L1-2 and in colon and that this could be reversed by PDTC directly acting on the ENS. This indicates that the NF-κB/iNOS pathway may be involved in the interaction between the spinal cord and colon. NF-κB is accepted to be an important upstream modulator of NO and iNOS production (Pahl, 1999). We found that CUMS exposure promoted nuclear translocation of NF-κB in spinal cord L1-2 and colon, which significantly increased the levels of iNOS-induced NO. Subsequently, a sustained high level of NO caused colon tissue damage. PDTC pretreatment inhibited nuclear translocation of NF-κB and down-regulated iNOS mRNA levels, which decreased the excessive iNOS-derived NO production induced by CUMS.

It is noteworthy that fluoxetine pretreatment reversed the depressive- and anxiety-like behaviors induced by CUMS and reduced the serum CORT level. However, it did not inhibit the trend of CUMS-induced slow weight gain after 4 weeks of exposure. Moreover, fluoxetine neither alleviated colon damage nor inhibited the overproduction of NO in spinal cord L1-2 and colon. Moreover, we also found that fluoxetine could not inhibit the production of iNOS induced by CUMS. However, there was no significant difference in NF-κB and iNOS levels when compared to the control rats. It is well known that fluoxetine usually exhibits its therapeutic effects 3–4 weeks later (Vaswani et al., 2003). Therefore, fluoxetine was able to exhibit antidepressant effects, whereas there was insufficient treatment time for it to inhibit the colon tissue injury and weight gain.

## Conclusion

In conclusion, our study demonstrated that CUMS was able to induce depressive and anxiety-like behaviors, accompanied by colon tissue damage, in rats. Furthermore, the NF-κB/iNOS pathway may be involved in the mechanism of CUMS-induced colon tissue injury, and this might provide new insight into a potential therapeutic strategy for the treatment of depression with GI dysfunction.

## Data Availability Statement

All datasets generated for this study are included in the article/supplementary material.

## Ethics Statement

The animal study was reviewed and approved by the Shandong University Animal Care and Use Committee.

## Author Contributions

LY and ML designed the experiments. HC, SN, and JD carried out the experiments. DW analyzed the experimental results. LY wrote the manuscript.

## Conflict of Interest

The authors declare that the research was conducted in the absence of any commercial or financial relationships that could be construed as a potential conflict of interest.
